# Dynamic Alloying of Steels in the Super-Deep Penetration Mode

**DOI:** 10.3390/ma15062280

**Published:** 2022-03-19

**Authors:** Yulia Usherenko, Viktors Mironovs, Sergey Usherenko, Vjaceslavs Lapkovskis, Andrei Shishkin

**Affiliations:** 1 Scientific Laboratory of Powder Materials, Faculty of Mechanical Engineering, Riga Technical University, 6A Kipsalas Str., Lab. 319, LV-1048 Riga, Latvia; viktors.mironovs@rtu.lv; 2 Powder Metallurgy, Welding and Materials Technology Department, Faculty of Mechanics and Technology, Belarusian National Technical University, Ya. Kolasa Str. 24, 220013 Minsk, Belarus; usherenko@gmail.com; 3 Rudolfs Cimdins Riga Biomaterials Innovations and Development Centre of RTU, Faculty of Materials Science and Applied Chemistry, Institute of General Chemical Engineering, Riga Technical University, Pulka 3, LV-1007 Riga, Latvia; andrejs.siskins@rtu.lv

**Keywords:** powder metallurgy, dynamic alloying, heat treatment, super-deep penetration, alloys, composite materials, mechanical properties

## Abstract

The dynamic effects observed in collisions represent a specific area of high-energy interaction located at the boundary of mechanics, hydrodynamics, shock wave physics, and alternating high-pressure regions. The paper shows that in the volume of a solid metal body, as a result of dynamic alloying by a high-speed stream of powder particles in the super-deep penetration mode (SDP), fiber structures of altering material arise, forming the framework of the composite material. The stream of powder particles in the metal obstacle following the path of least resistance and the impact of shock waves on particles results in a volumetric framework from the products of interaction between the injected and matrix materials. When using SDP, defective structural elements (channeled)—germs of reinforcing fibers arise. At the subsequent heat treatment, there is an intensive diffusion. The growth process of reinforcing fibers shifts to higher temperatures (as compared to the standard mode), leading to an increase in the bending strength of the fiber material up to 13 times for W6Mo5Cr4V2 high-speed tool steel. As a result of the completion of the growth of reinforcing fibers in the volume of the W6Mo5Cr4V2 high-speed tool steel, the material’s bending strength in 1.2 times is realized. Simultaneously, it provides an increase of wear resistance 1.7–1.8 times.

## 1. Introduction

The development of modern engineering and technology in new materials creation, environmental protection, and power engineering is inseparably connected with the solution of a whole set of the most important fundamental problems. One of these tasks is the creation of efficient approaches to creating new composite materials with a high level of performance properties, reduction of mining, decrease in the need for non-renewable critical raw materials, lowering the energy intensity of production.

Resource demands will continue to increase due to global population growth, industrialization, increasing demand from developing countries, and the transition to climate neutrality using metals, minerals, and biotic materials in low-emission technologies and products [1]. Reliable and sustainable supplies of critical raw materials for key critical technologies and strategic sectors are necessary for achieving climate neutrality. Therefore, various options are being considered to ensure an uninterrupted supply of ferroalloy elements. For example, methods for extracting raw materials from mining waste and scrap metal are being developed and improved [2]. Another important direction is to increase the efficiency of alloying elements use, tool protection using coatings, cooling, development of the more efficient shape of products, reduction of ferroalloy elements use due to lower concentration in steels [3].

The shock wave method is a powerful and unique tool for studying the properties of materials at extremely high strain rates with well-controlled loading conditions. Experiments with shock waves are characterized by a wide range of attainable pressures and temperatures and extremely high rates of change. These circumstances open up unique opportunities for research in the physics of phase and polymorphic transformations, fracture physics, strength and plasticity, and materials science [4]. Furthermore, high-energy processing features make it possible to significantly change the physical and chemical properties of materials [5] and, consequently, the operational characteristics of tools and parts. During a super-deep penetration (SDP), numerous factors acting simultaneously on the material are realized.

### 1.1. Dynamic Alloying in the SDP Mode

SDP is a complex physical phenomenon. A split-second stream of powder particles with a fraction less than 200 microns, accelerated to speeds of 700–3000 m·s^−1^, penetrates the solid metal body at depth in tens, hundreds mm. At the same time, the high and ultra-high pressure (0.2–20 GPa), intensive deformation, local heating, friction is occurred [6].

Dynamic alloying in the SDP mode is characterized by the simultaneous action of various physical factors on the material (high pressure, significant pressure gradients inside a solid metal body, zones of intense tension and compression, temperature, radiation). Such a complex effect occurs in the time interval of 10^−9^–10^−4^ s, which significantly changes the conditions of heat and mass transfer. The loading intensity is so high that destruction or irreversible changes are possible in the body on which it acts.

Under pulsed and shock impacts, disturbance regions with a complex stress-strain state and metastable structural elements can form in the material [7]. The structure of the matrix material in the areas of ultra-high pressure is ground up to complete amorphization. These areas intertwine with other matrix regions, powder particles. As a result, forming a polycrystalline reinforcing framework and an anisotropic composite material [8]. This physical phenomenon occurs only in a closed system. Unlike classical alloying, SDP introduces alloying elements into an already solid body and to a greater depth than when using surface hardening and ion implantation methods [7,8,9].

### 1.2. SDP Models

The shock wave technique is a powerful tool for studying the properties of materials at extremely high strain rates with well-controlled loading conditions. An additional factor in the impact in the SDP mode is the combination of shockwave effects with high-speed particles. It is also relevant for space material science to study the effects of cosmic dust streams on spacecraft components.

Several papers [10,11,12,13] are devoted to reviewing the results in this area. In [10,11], simulation and analysis of the possibility of using an explosive accelerator to simulate the impact of cosmic dust stream on spacecraft materials in ground conditions. Refs. [12,13,14,15,16] presented modeling and experimental data on the interaction of high-speed bodies and particles with spacecraft materials. The authors of [17,18,19] present methods of high-speed dust particle streams acceleration.

Several authors have proposed variants for modeling the interaction of particles during high-speed impact with an obstacle [18,19,20,21,22,23,24,25].

The matrix material under dynamic processing acquires quasi-liquid properties, which leads to the effects of local turbulence, local deformation, local alloying, local heating, and local supercooling. The authors of the publications [26,27,28,29,30,31,32] studied the effect of dynamic action on several materials, which showed a significant effect of dynamic action on the structure, chemical composition, and properties of the processed materials. The authors of papers [33,34,35,36,37] investigated the effect of high-energy exposure combined with the impact on the target by a stream of high-speed particles.

In recent years, many authors have carried out experimental and numerical studies of dynamic effects on various materials, confirmed the promise of research on complex, high-energy effects on materials, and proposed some models for predicting the behavior of materials. At the same time, numerous models explaining the realization of dynamic alloying during SDP are based on qualitatively different physical mechanisms that contradict each other.

Most of the models offer an explanation of SDP by developing mechanisms of interaction of particles or particle stream with an obstacle with minimization of energy input and, consequently, interaction, i.e., with the impact kinetic energy efficiency close to or equal to 100%. However, there is still no model that would fully describe all of the effects inherent in the SDP. If to quote the calculations in all models, the experimental depth cannot be achieved. Furthermore, wave effects, which have a primary role, have not been considered. Due to the multi-parameter nature of the problem and the lack of a unified model, it is not easy to predict the properties of the resulting materials.

### 1.3. Powder Particles Acceleration Methods

Experimental study of processes realized during high-speed impact requires the selection of appropriate accelerators to accelerate microparticles of a particular mass to specified velocities. Explosive, light-gas, and electromagnetic accelerators of various types are widely used to accelerate macroparticles [13].

The magnetic-pulse method is used for throwing bodies with a mass of 0.1–500 g. It is carried out at speeds of 30–1000 m·s^−1^. The principle of converting electric energy stored in the accumulator into a powerful pulsed magnetic field, which affects the processed material in a strictly dosed form, is used in magnetic-pulse material processing technology [38].

Light-gas guns, two-stage, in the first stage forming a gas phase, provide throwing with a maximum velocity of up to 7000 m·s^−1^. The pressure in the gas front at maximum when fired is up to 1 GPa.

Explosive methods are the most effective, providing acceleration of bodies due to initiation of explosives with detonation velocity up to 9000 m·s^−1^ and pressure in the wavefront of 10–100 GPa. Explosive accelerators are inexpensive and straightforward and have found widespread use in practice. Acceleration does not depend on the material of the accelerated jet (conductive, non-conductive). When they are used, the stream of microparticles is formed when the explosive charge compresses the container with the powder particles [37].

The main advantages of the explosive acceleration method are the high acceleration velocities of the powder stream and the amount of energy applied to the accelerated stream. A disadvantage is a low number of pulses. The main advantages of the electromagnetic acceleration method are a more comprehensive range of particle sizes and a high number of pulses. A significant advantage of magnetic pulse accelerators is a higher level of staff safety.

**Figure 1:**
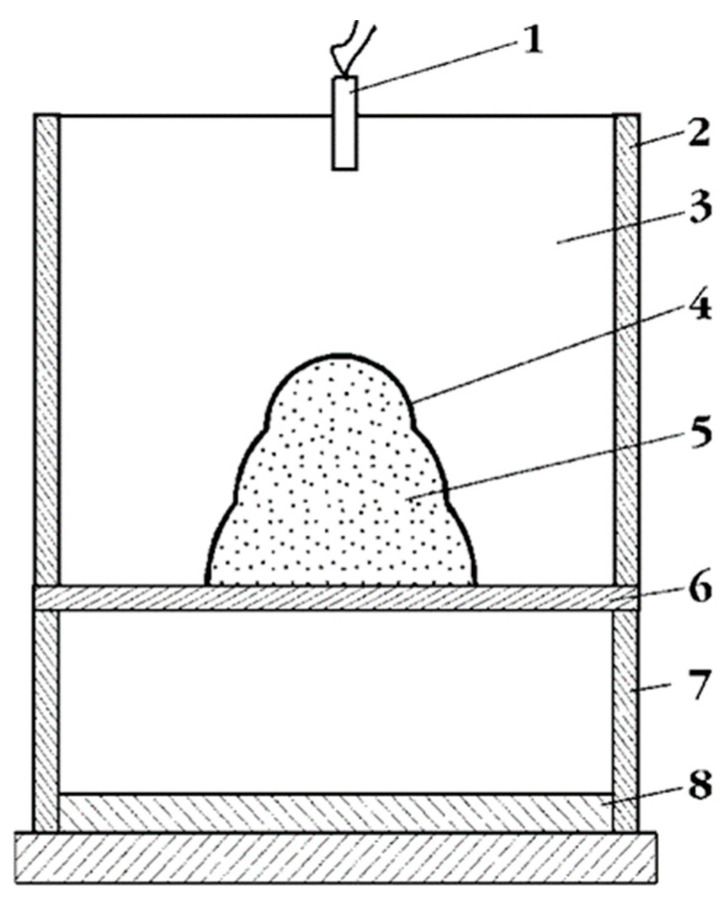
Outdoor explosive accelerator [39]: 1—initiator, 2—facing, 3—charge, 4—cumulative lens, 5—powder mixture, 6—plate-cartridge base, 7—adjusting support, and 8—sample.

In order to conduct experiments on dynamic alloying of steel samples, an explosive accelerator (Figure 1) described in [39] was used. It is based on the scheme of an explosive accelerator with a cumulative lens. In this case, the high-energy stream’s velocity is 300–3000 m·s^−1^.

## 2. Materials and Methods

**Figure 2:**
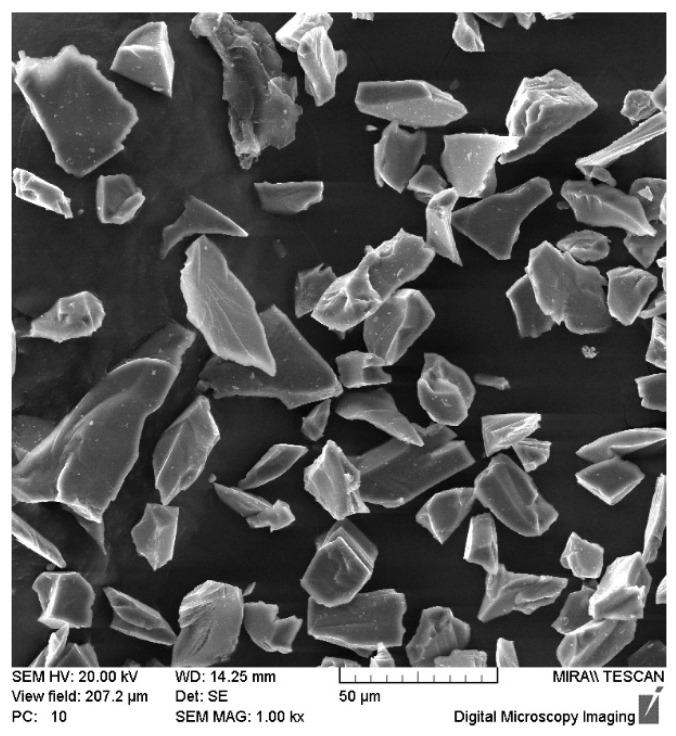
The SiC powder structure.

Dynamic processing in SDP mode was carried out in the following conditions [39]: average particles speed 3000 m·s^−1^, exposure time ~400 microseconds, the material of billets: carbon steel of ordinary quality St3 (0.14–0.22% C, DIN St37-2), high-quality structural carbon steel 10 (0.07–0.14% C, DIN C10), W6Mo5Cr4V2 (0.82–0.90% C, 5.5–6.5% W, 4.8–5.3% Mo, 3.8–4.4% Cr, 1.7–2.1% V) high-speed tool steel (DIN HS6-5-2). Cylindrical samples, diameter 30 mm, length 100 mm. The material of powder particles stream—SiC powder (Figure 2 and Table 1) (for carbon steel of ordinary quality St3, high-quality structural carbon steel 10), SiC + Ni powder mix (W6Mo5Cr4V2 high-speed tool steel), and SiC + TiCN powder mix (W6Mo5Cr4V2 high-speed tool steel), 50–63 microns. The mixtures of powders were mixed for 60 min. The ratio of SiC + Ni and SiC + TiCN mixtures is 50/50%.

### 2.1. Scanning Electron Microscopy

Structures were studied by scanning electron microscope (SEM) MIRA TESCAN(Tescan, Czech Republic), MIRA3 TESCAN(Tescan, Czech Republic), Hitachi S-4800 (Hitachi, Japan).

The samples preparation technique includes cutting, polishing, and etching in 4% nitric acid.

Scanning electron microscopes are equipped with secondary electron detectors (SE) and backscattered electron detectors (BSE), which allow the examination of samples in two modes. In the secondary electron (SE) mode, the contrast in the image is created by the reflection of the electron beam from the surface of the sample. In the case of a backscattered electron detector (BSE), the contrast in the image is created by the difference of the “averaged” atomic numbers. The type of detector used, and other imaging parameters are indicated on the information line at the bottom of each frame.

Micro-X-ray spectral analysis of samples was performed both along the line (concentration curves of element distribution) and along the area (characteristic X-ray emission maps). Characteristic X-ray imaging was used to study the distribution of elements on the surface.

On the concentration curves of elements distribution along the y-axis, the intensity of X-ray radiation of elements of the sample is indicated, which is proportional to the concentration.

A study of structural elements on SEM images of steel samples before and after treatment was performed to determine the dependence of the forming microstructure on the impact parameters. The main topographic element arising during the impact of a high-speed microparticle with the matrix metal is a channel. When considering the channel in a cross-section perpendicular to the stream introduction, it is determined that the channel shape is close to a circle. In the sample volume, the channels form the structure of a porous body.

The fraction of altered material was used as an experimental criterion for evaluating the structural changes occurring in the material. The altered material fraction is the volume of material in which structural rearrangement has occurred in the SDP mode. This activated material is retained in the barrier after dynamic mass transfer caused by significant pressure field gradients.

Subsequent etching results in activation and etching of this material with a different etching rate compared to that of the matrix material, allowing this visible porosity value, C (%), to be assessed using electron microscopy techniques. In addition, determination of parameters such as d_k_—the average visible diameter of the channel (μm) was also performed. The study was carried out using images with a magnification of 10,000 and 15,000.

### 2.2. Hardness Measurement

The hardness of unhardened steel was measured by the Brinell method (HBW). Ball diameter D = 10 mm, nominal value of test force F = 29,420 N (ISO 6506-1:1999).

The hardness of the hardened samples was measured with a diamond cone on a C scale, Rockwell method (HRC). Diamond cone with total force F = 1.471 kN (ISO 6508-1:2016). Samples were cut along the depth of the sample in 20 mm increments.

### 2.3. The Mechanical Properties Measurements

The wear resistance of the samples was measured on a friction machine (BNTU, Belarus) according to the two-body abrasion scheme. The load is 2 kg, samples size is 8 × 8 × 50 mm. Wear test specimens were cut from workpieces processed in the SDP mode. The tests were carried out along the section along with the depth of the workpieces. The weight and volume of each sample were determined prior to testing. Then, every hour after abrasion (friction), each test sample was weighed, its residual weight was determined, and its residual volume was measured. In this way, changes in the specific gravity of the material of the samples over depth were determined.

Bend tests were performed according to ISO 7438:2016. Tensile tests were performed at room temperature following ISO 6892-1:2009. Circular notched specimens were used to ensure that the explosion-treated material could be characterized in depth. The notch angle was 60°, and its depth was 0.5 mm.

Tests for impact toughness (dynamic bending) are carried out with oscillography of fracture process on a rotary press, following ISO 148-1. Impact velocity is 4.5 m·s^−1^, sample dimensions are standard (10 × 10 × 55 mm). No incision was made from the surface because the materials tested are prone to brittle fracture.

The numerical values in the data tables and points in the graphs represent the arithmetic mean of the parameters estimated from a sample of at least five experimental results.

## 3. Results

### 3.1. Changes in the Structure of Carbon Steels after Dynamic Alloying in the SDP Penetration Mode

It is assumed that the result of a high impulse pressure on steel is the compression of the substance. Such a physical process leads to the cumulation of energy of the interacting substances. Nowadays, it is established that increasing the density of the high-pressure phase is not a general rule [4,40]. Phase transitions and density changes significantly impact the mechanical properties of steel [29].

**Figure 3:**
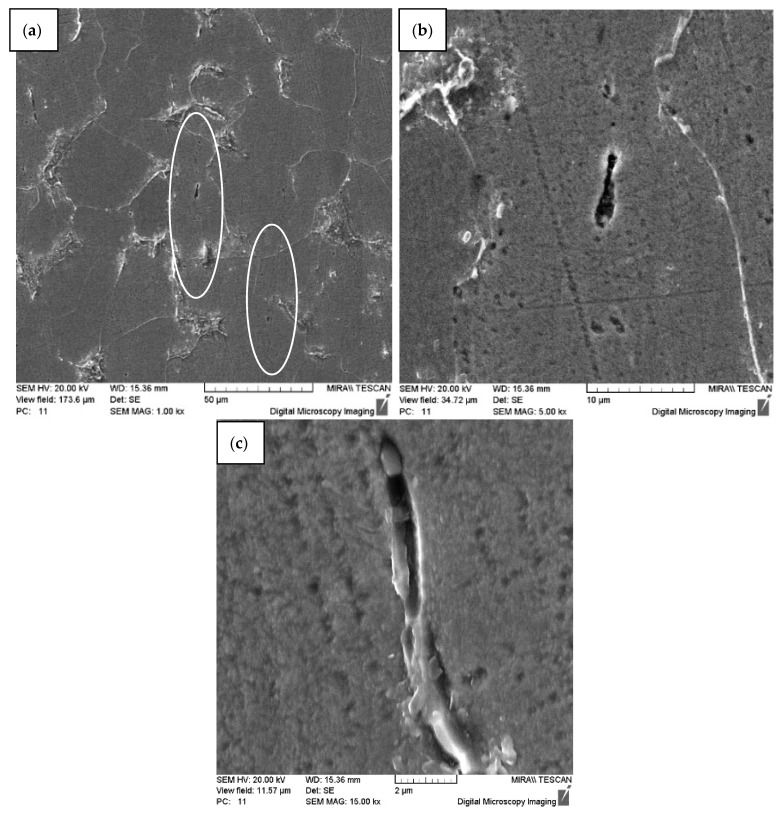
The structure of steel St3 after the dynamic alloying in the SDP mode. Longitudinal direction of the section (along the direction of particle stream): (**a**) Microstructure of steel St3 after the dynamic alloying in the SDP mode with channels (in circles); (**b**,**c**) channel element (fiber).

**Figure 4:**
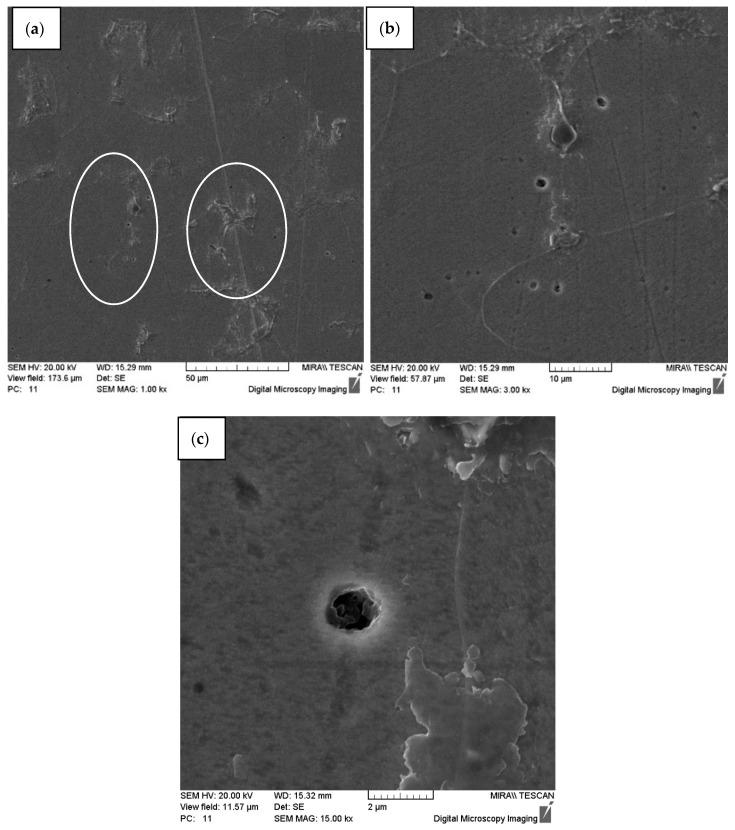
The structure of steel St3 after the dynamic alloying in the SDP mode. Transverse direction of the section (across the direction of particle stream): (**a**) Microstructure of steel St3 after the dynamic alloying in the SDP mode with channels (in circles); (**b**,**c**) channel elements (fibers).

**Figure 5:**
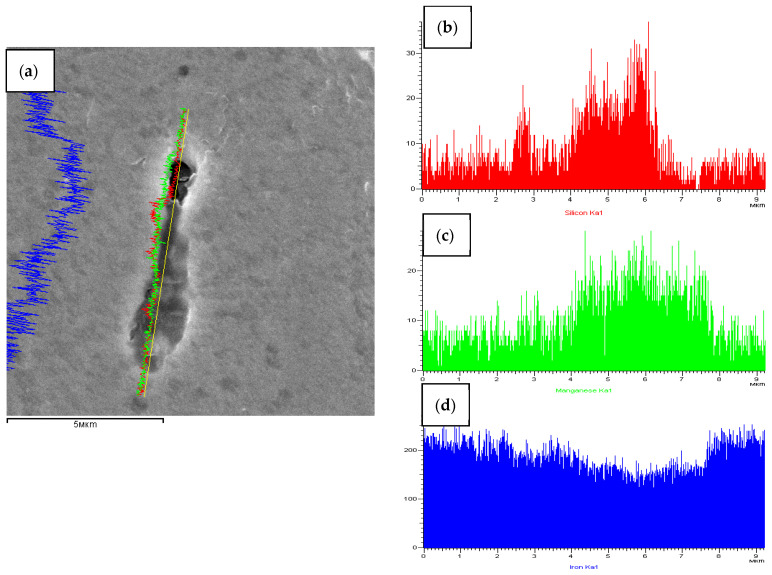
The qualitative analysis of channel element of the structure of steel St3 after the dynamic alloying in the SDP mode: (**a**) Channel element (fiber) in the structure steel St3 after the dynamic alloying in the SDP mode; concentration curves of elemental distribution: (**b**) Silicon, (**c**) Magnesium and (**d**) Iron.

**Figure 6:**
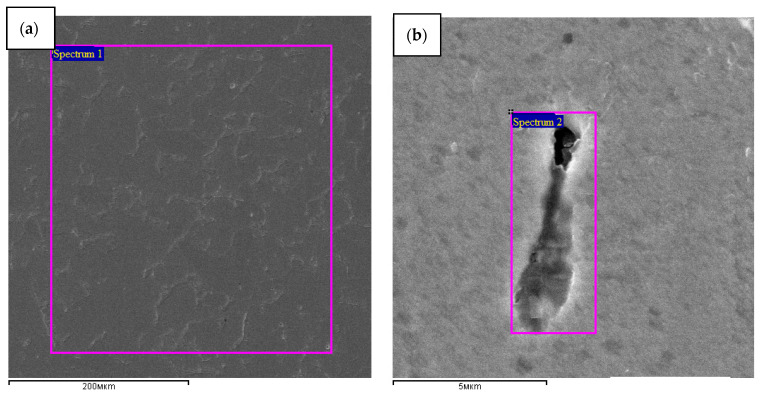
Zones of composition analysis (**a**) the initial steel 3 and (**b**) channel element(fiber) section in steel 3 after dynamic alloying with SiC powder in the SDP mode.

**Figure 7:**
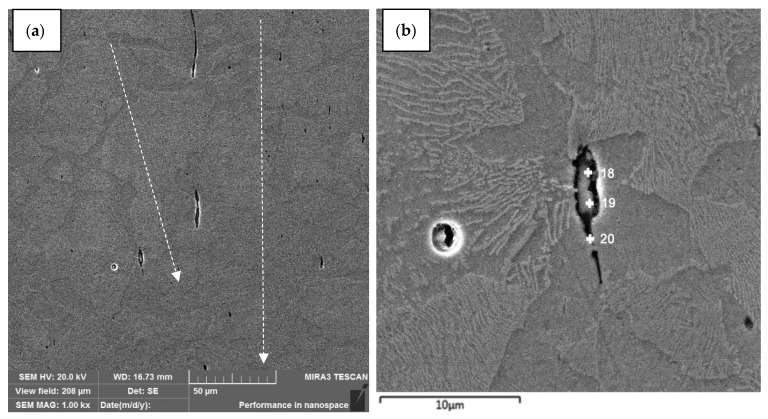
The structure of steel 10 after the dynamic alloying in the SDP mode. Longitudinal direction of the cross-section (along the direction of particle stream: (**a**) Microstructure of steel 10 after the dynamic alloying in the SDP mode with channels(fiber) and (**b**) channel element (fiber) with elemental analysis points.

Research on the effect of dynamic alloying by streams of SiC powder particles on samples of carbon steel of ordinary quality St3 and high-quality structural carbon steel 10 has shown the treatment effect on the structure of steels with the formation of characteristic channel elements (fibers) (Figure 3, Figure 4, Figure 5, Figure 6 and Figure 7). In the solid metal body volume after SDP, fiber structures from the residual defective material form a framework. The fibers are highlighted out in the form of track formations (revealed by etching) in the section of the cross-section (Figure 3 and Figure 4).

The effects realized in carbon steels under dynamic impact allow the creation of anisotropic composite materials. Changing the structure of the material should affect the properties of the resulting material.

Figure 5 and Figure 6 and Table 2 show the quantitative and qualitative microanalysis results of the composition of the channel element residues. The detection of channels is performed by the difference in the etching rate with the matrix material. Therefore, part of the channel element material can be etched at the preparatory stage. However, residual SiC powder was detected (Figure 6b, Table 2).

Figure 7 shows a part of the channel formed by the interaction of the high-speed stream of powder particles and the matrix material (high-quality structural carbon steel 10). Residual SiC powder was detected (Table 3). The dashed line indicates the direction of the channels. Due to the curvilinear nature of the movement of powder particles in the matrix material, the entire channel, which is formed during their movement, is not in the same plane of the cross-section.

It is not enough to perform the process of dynamic alloying with powder particles in the SDP mode to increase mechanical properties. The state of the synthesized strengthening material in the reinforcing zones is metastable because the processes of structural rearrangement remain incomplete through the realization of SDP in the time of 10^−8^–10^−4^ s. Therefore, following the technological process scheme, a heat treatment process in which diffusion processes and structure rearrangement are completed is necessary to produce massive tool composite material.

The study of changes in the properties of steels more complex in structure and has a broader application in the industry will show the possibility of regulating the properties of steels by dynamic alloying in the mode of SDP penetration.

### 3.2. Dynamic Alloying of High-Speed Steel W6Mo5Cr4V2 in the SDP Mode

Consider the introduction of powders based on silicon carbide with nickel and titanium carbonitride with nickel as a technological option in the W6Mo5Cr4V2 high-speed tool steel. Silicon carbide and titanium carbonitride are tough and light materials. Nickel metal powder is used as an additive in the mixtures.

Marking the W6Mo5Cr4V2 steel tested samples: initial sample—No. 1, W6Mo5Cr4V2 steel treated with SiC + Ni mixture—No. 2, W6Mo5Cr4V2 steel treated with TiCN + Ni mixture—No. 3.

After dynamic alloying standard heat treatment was performed: T hardening = 1220 °C, T tempering = 560 °C (3 times 560 °C for 1 h).

**Figure 8:**
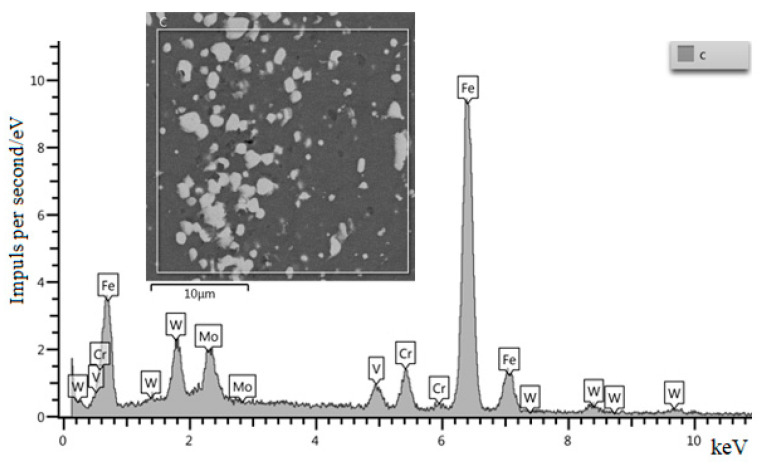
Qualitative analysis of the initial W6Mo5Cr4V2 steel (without dynamic treatment).

**Figure 9:**
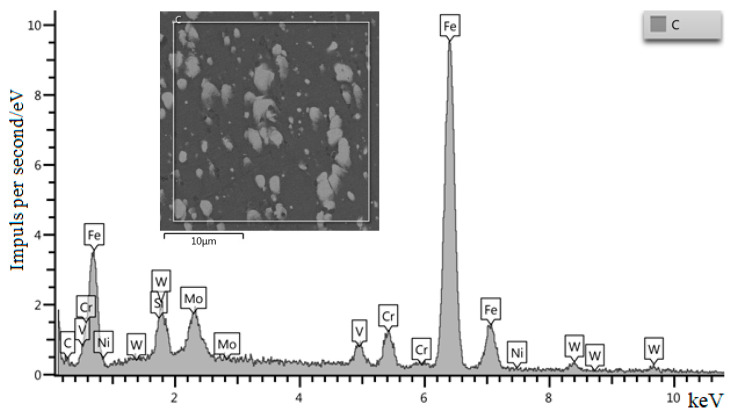
Qualitative analysis of the chemical composition of the W6Mo5Cr4V2 steel after dynamic alloying with SiC + Ni powder.

Figure 8 shows the qualitative analysis of the composition of the initial W6Mo5Cr4V2 steel. Figure 9 shows the qualitative analysis of the composition of W6Mo5Cr4V2 steel after dynamic alloying in the SDP mode by the stream of SiC + Ni particles. The analysis shows the presence of nickel in the structure of the treated steel.

**Figure 10:**
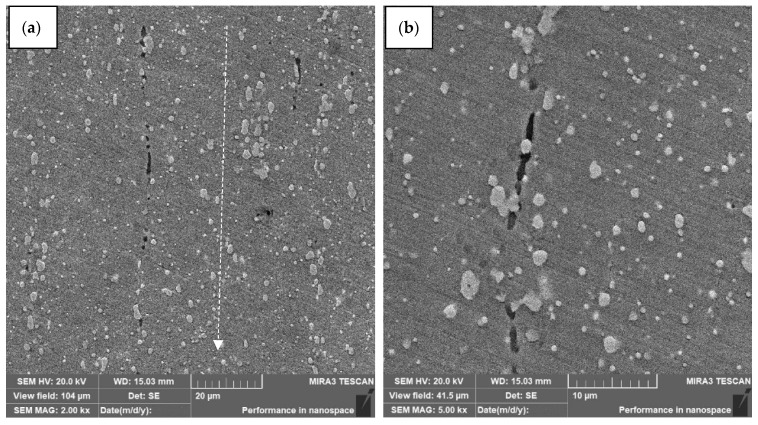
The structure of W6Mo5Cr4V2 steel with channel elements after dynamic alloying in the SDP mode by SiC + Ni stream and subsequent hardening. (**a**) Microstructure of W6Mo5Cr4V2 steel with channels; (**b**) channel elements (fibers).

Figure 10 shows the structure of W6Mo5Cr4V2 steel with channel elements after dynamic alloying in the SDP mode by SiC + Ni stream and subsequent hardening.

**Figure 11:**
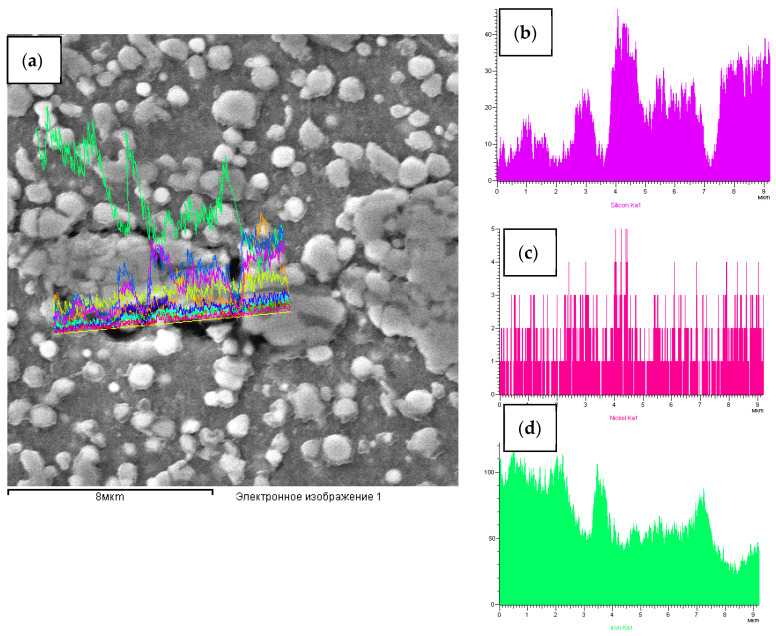
The qualitative analysis of channel element of the structure of W6Mo5Cr4V2 steel after the dynamic alloying in the SDP mode: (**a**) Channel element(fiber) in the structure W6Mo5Cr4V2 steel after the dynamic alloying in the SDP mode; concentration curves of elemental distribution: (**b**) Silicon, (**c**) Nickel and (**d**) Iron.

Figure 11 and Table 4 show the qualitative and quantitative analysis of the channel element. The analysis confirms the presence of powder stream SiC + Ni residues in the channel element.

The study of the hardness of W6Mo5Cr4V2 steel (Table 5) showed that dynamic alloying in the SDP mode with SiC + Ni powders led to a decrease in the hardness of unhardened steel by 2.7%, and after hardening by 3.8%. At the same time, the use of a mixture of powders TiCN + Ni provided an increase in the hardness of W6Mo5Cr4V2 steel by 5.6% and hardened steel by 4.8% compared to the initial hardened W6Mo5Cr4V2 steel.

The change in mechanical properties under dynamic alloying [35,41,42,43] indirectly points to a change in the material’s physical properties, and the peculiarities of the dynamic alloying process in the SDP mode should increase the anisotropy of the material being processed.

**Figure 12:**
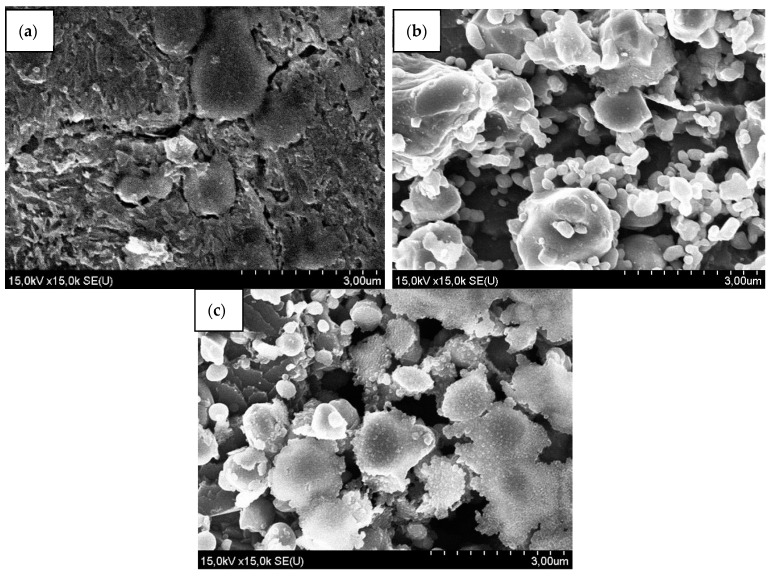
The structure of W6Mo5Cr4V2 steel (**a**) W6Mo5Cr4V2 steel microstructure without dynamic alloying after hardening, (**b**) W6Mo5Cr4V2 steel microstructure after dynamic alloying in the SDP mode by TiCN + Ni stream and hardening, (**c**) W6Mo5Cr4V2 steel microstructure after dynamic alloying in the SDP mode by TiCN + Ni stream, forging and hardening.

Consider the structures of the hardened W6Mo5Cr4V2 steel without dynamic alloying, W6Mo5Cr4V2 steel, which were subjected to dynamic alloying in the mode of SDP by the stream of powder particles of TiCN and Ni and hardened, and W6Mo5Cr4V2 steel samples, which in addition to dynamic alloying were hardened and forged (Figure 12).

The structure of the hardened W6Mo5Cr4V2 steel without dynamic alloying (Figure 12a) has significant differences from the structure of steel after dynamic alloying (Figure 12b) and dynamic alloying and forging (Figure 12c). The etching rate of the treated samples increased sharply. In addition, it should be noted that the change in the shape of grains of the sample was forged after SDP (Figure 12c).

For further studies, the samples treated in the SDP mode with a mixture of TiCN and Ni powders were selected. After dynamic alloying in SDP mode, heat treatment was performed: T hardening = 1200 °C, 1210 °C, 1220 °C, T tempering = 520 °C, 530 °C, 540 °C, 550 °C, 560 °C, 570 °C, 580 °C, 590 °C, 600 °C (3 times for 1 h).

## 4. Discussion

### 4.1. Changes in the Structure of W6Mo5Cr4V2 Steel at Different Processing Variants

In the volume of the solid metal body as a result of this dynamic alloying, fiber structures arise, forming the framework of the composite material. Reinforcement in the transverse direction arises due to the rotation of powder particle streams at an angle from the longitudinal direction to the sidewalls of the metal barrier. As a result of the movement of the stream of powder particles in the metal barrier along the path of least resistance and impact of shock waves on the powder particles, a volumetric framework is formed from the products of interaction of the injected and matrix materials.

**Figure 13:**
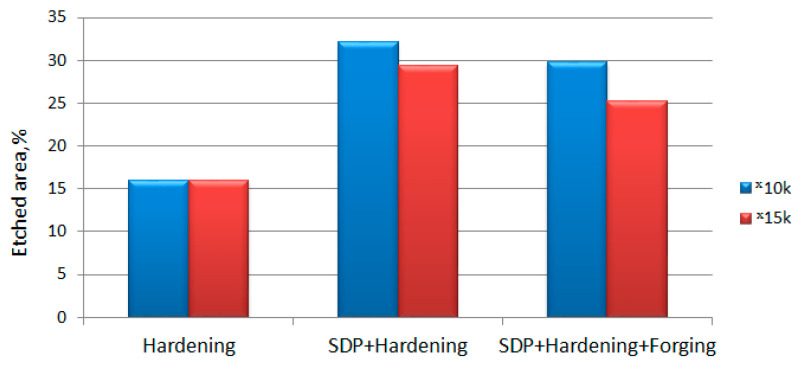
Area of activated and etched material in cross-section of specimens made of W6Mo5Cr4V2 steel (magnification 10,000, magnification 15,000).

It is worth comparing the different types of effects on W6Mo5Cr4V2 high-speed tool steel. A quantitative analysis of the structural changes in the activated zones showed (Figure 13) that the hardened material without dynamic treatment has the lowest etching rate (16% of the total scan area). In comparison, the maximum etching rate is shown by the SDP-treated + hardening (29–32%), as well as the additionally treated (SDP), forged, and hardened (25–29%) samples.

**Figure 14:**
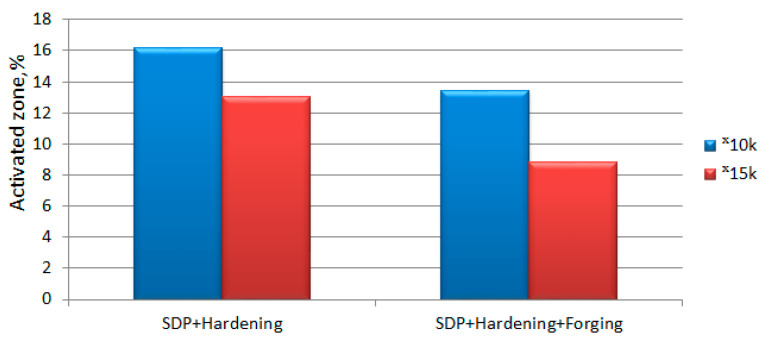
Activated zone area of treated samples of W6Mo5Cr4V2 steel excluding the influence of the material of the initial sample (magnification 10,000, magnification 15,000).

For further analysis, the etching rate value of the hardened material without dynamic treatment was excluded from the parameters of the treated samples (Figure 14), which allows us to exclude the influence of common factors of the initial W6Mo5Cr4V2 steel and heat treatment. It should be noted that the area of the activated zone is higher in the samples after treatment in the SDP + hardening mode as compared to the samples that underwent additional forging and hardening. The difference is ~3%. This effect should be attributed to the influence of processing, which leads to a decrease in the effect of SDP activation by homogenizing the structure of the processed samples.

**Figure 15:**
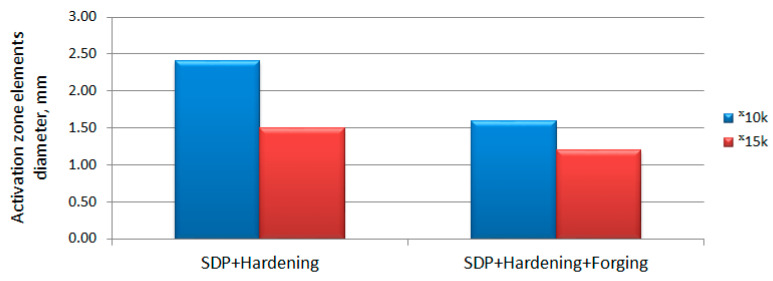
Diameter of activation zone elements of W6Mo5Cr4V2 steel samples (magnification 10,000, magnification 15,000).

Consider the value of the elements of the activated zones. As follows from Figure 15, the value of the elements of the activated zones is higher in the samples treated in the SDP mode. The ratio of the average size of the defective (activated) zones of the W6Mo5Cr4V2 steel treated in the SDP mode and the same steel after an additional forging operation is 1.25–1.5 times. Thus, forging leads to homogenization of the structure and, simultaneously, refinement of its elements.

### 4.2. Changes in Mechanical Properties of W6Mo5Cr4V2 High-Speed Tool Steel, Activated in the SDP Mode by Controlling the Modes of Subsequent Heat Treatment

The metastable state of the framework material, which is explained by the short time of SDP realization (10^−8^–10^−4^ s) and the remaining incomplete diffusion processes, indicates the possibility of increasing the mechanical properties of the composite material by subsequent heat treatment [44].

Therefore, producing a massive tool composite material requires a subsequent heat treatment or thermo-mechanical treatment process in which the restructuring of the structure is completed.

**Figure 16:**
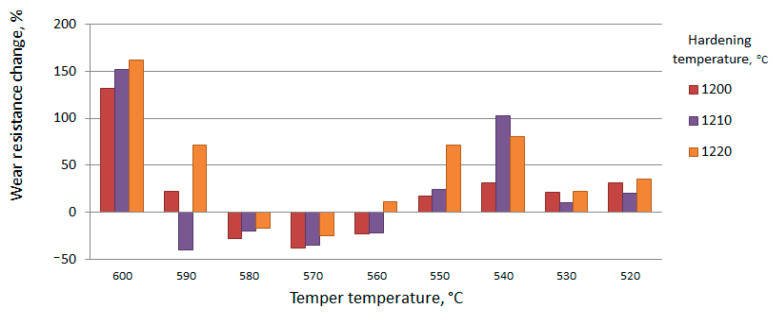
Effect of heat treatment modes on the change in relative wear resistance (%) (W6Mo5Cr4V2←TiCN + Ni).

**Figure 17:**
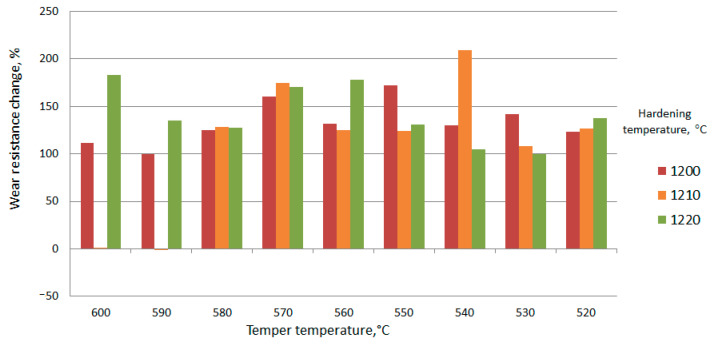
Effect of heat treatment modes on the change in relative wear resistance (%) (W6Mo5Cr4V2←TiCN + Ni).

For various cutting tools, the main criterion is the wear resistance of the tool material. Therefore, the relative wear resistance will be used for the primary evaluation of the hardened material. The composition of the introduced substance essentially influences the change of relative wear resistance in the whole range of heat treatment regimes. Consider the change in the relative wear resistance of the steel matrix hardened by the s of TiCN particles (Figure 16 and Figure 17).

From the above results, we can conclude that it is necessary to coordinate the modes of each type of treatment. The optimal heat treatment mode for the matrix material is hardening at 1220 °C and three-times tempering at 560 °C. The area of increased wear resistance corresponds, as a rule, to the increased temperatures during hardening and tempering. The change in wear resistance is associated with an increase in the volume fraction of the strengthening phase. The volume of strengthening phases changes due to diffusion processes during heating.

Consider the change in the bending strength of composite material after treatment of steel workpieces with a mixture of powders in the SDP mode. Basic assumptions: the fibers and the matrix are deformed in the composite in the same way as in their separate tests; all the fibers have the same tensile strength values and collapse at the same strain. Considering these assumptions, the rule of mixtures determines tensile strength of composite materials (additivity) σ_∑_ = σ_f_⋅V_f_ + σ_m_ (1 − V_f_), where σ_∑_—bending strength of a composite, σ_f_—bending strength of fibers, σ_m_—bending strength of the matrix, V_f_—volume fraction of fibers.

**Figure 18:**
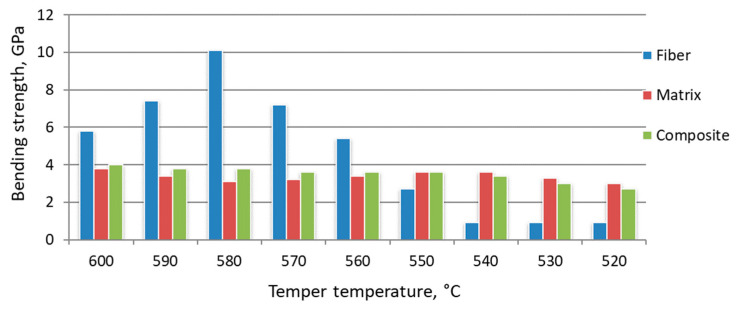
Effect of tempering temperature on the characteristics of composite material based on W6Mo5Cr4V2 steel, hardened at 1290 °C.

**Figure 19:**
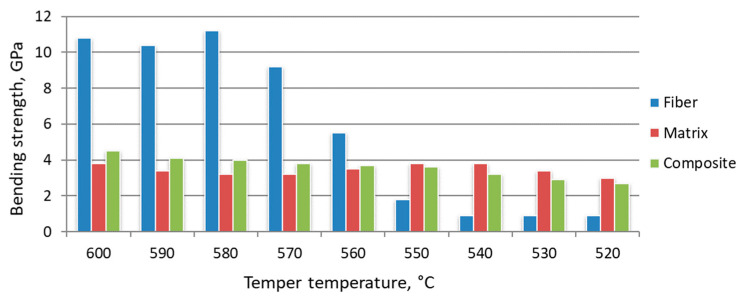
Effect of tempering temperature on the characteristics of composite material based on W6Mo5Cr4V2 steel, hardened at 1210 °C.

**Figure 20:**
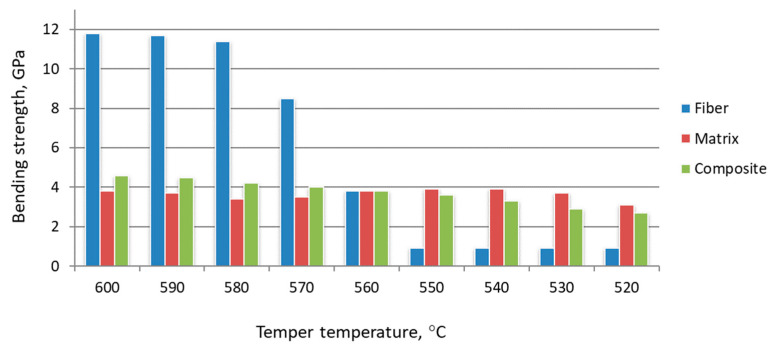
Effect of tempering temperature on the characteristics of composite material based on W6Mo5Cr4V2 steel, hardened at 1220 °C.

The share of fiber structure in the volume of the composite material was determined to estimate the strength of fiber structures σ_f_. As a rule, the share of zones “influence” of fiber structure in composite metal material is set at 10% of volume, excluding overestimation of σ_f_. Bending strength and structure parameters after hardening from different temperatures are considered in Figure 18, Figure 19 and Figure 20.

Tempering at temperatures of 550 °C and below, the change in the bending strength of the reinforcing and matrix material is negative. The strength of the activated zones at the same heat treatment modes is less than that of the matrix. It is assumed that the growth process of strengthening phases at these tempering temperatures has not been completed. In the activated zone, the stage of grain growth is preceded by the manifestation of new growth points. Accordingly, the fiber diameter at tempering temperatures below 550°C becomes larger than at higher temperatures. The fiber material with incomplete structural rearrangement is friable. At this stage of heat treatment, the presence of activated zones in the volume of the steel billet leads to a decrease in the strength characteristics of the product. Comparison of the results, presented in Figure 16, Figure 17, Figure 18, Figure 19 and Figure 20, shows that the growth of strengthening fibers shifts to higher temperatures, thus increasing to bending strength of fiber material 13 times. The strength of the grown fibers is comparable to that of ceramic and metal whiskers. It provided a 1.2 time increase in the bending strength of the new tool material. At the same time, it provided an increase in wear resistance by 1.7–1.8 times.

## 5. Conclusions

Research on the effect of dynamic alloying by streams of SiC powder particles on samples of carbon steels has shown the treatment effect on the structure of steels with the formation of characteristic channel elements (fibers) (Figure 3, Figure 4, Figure 5, Figure 6, Figure 7, Figure 10 and Figure 11). In the solid metal body volume after SDP, fiber structures from the residual defective material form a framework. The fibers are highlighted out in the form of track formations (revealed by etching) in the section of the cross-section. Changing the structure of the material should affect the properties of the resulting material.

The effects realized in steels under dynamic impact in the SDP mode allow the creation of anisotropic composite materials. In the solid metal body volume after SDP, fiber structures from the residual defective material form a framework. It is shown that the use of high-energy processing features makes it possible to significantly change the level of mechanical properties of steels and, accordingly, the operational characteristics of tools and parts.

It has been also established that to increase the mechanical properties of steel products, it is not enough to perform the penetration process with powder particles in the SDP mode. The state of the synthesized strengthening material in reinforcing zones is metastable because the processes of structural rearrangement remain incomplete through the realization of SDP in the time of 10^−8^–10^−4^ s. A further heat treatment process is necessary during which the diffusion processes and structural rearrangement are completed.

Based on the analysis of the processing variants of high-speed steel: standard heat treatment, SDP + standard heat treatment, SDP + forging + standard heat treatment, the arising structural differences are shown.

The effect of heat treatment modes after high-energy impact in the SDP mode was also investigated. The performed studies allow to perform the following main conclusions:

The use of an additional forging operation of W6Mo5Cr4V2 high-speed tool steel resulted in a 1.25–1.5 times smaller average size of the zone of activated (defective) material compared to the average size of the zone of defective (activated) steel treated in the SDP mode.

During the hardening of materials treated in the SDP mode, it is necessary to harmonize the modes of each type of treatment. Since using SDP defective structural elements—nuclei of reinforcing fibers appear. At the subsequent heat treatment, there is an intensive diffusion. Thus, the growth of reinforcing fibers shifts to higher temperatures (in comparison with the standard mode), leading to an increase in the bending strength of the fiber material by 13 times.

As a result of the completion of the growth of reinforcing fibers in the volume of tool W6Mo5Cr4V2 high-speed tool steel, the increase in bending strength of the material of 1.2 times is realized. Simultaneously, it provides an increase of wear resistance 1.7–1.8 times.

## Figures and Tables

**Table 1:** Phase composition of SiC particles.

Formula	Phase Name	Content (%)
SiC	Moissanite-6H	86
C	graphite	6
C60	fullerite	8

**Table 2:** Analysis of the composition of the initial steel 3 and channel element section in steel 3 after dynamic alloying in the SDP mode.

Spectrum	Elemental Concentration, %				
	Si	S	Mn	Fe	Sum
1	0.3	0.1	0.7	98.9	100.0
2	1.2	0.1	1.2	97.6	100.0

**Table 3:** Analysis of the composition of the channel element site in steel 10 after dynamic alloying in the SDP mode.

Point	Elemental Concentration, %				
	Si	S	Mn	Fe	Sum
18	1.12	20.02	37.67	41.19	100.0
19	1.20	25.46	52.75	20.59	100.0
20	1.32	0.00	3.35	95.33	100.0

**Table 4:** The quantitative analysis of channel element of the structure of W6Mo5Cr4V2 steel after the dynamic alloying in the SDP mode with SiC + Ni powder.

Point	Elemental Concentration, %							
	Si	V	Cr	Fe	Ni	Mo	W	Sum
1	0.5	0.8	6.3	89.5	0.5	1.3	1.1	100.0

**Table 5:** The W6Mo5Cr4V2 steel hardness.

Sample	HBW Average before Hardening	HRC Average before Hardening	HBW Average after Hardening	HRC Average after Hardening
Initial	223	21.0	635.1	61.7
SiC + Ni	217 (−2.7%)	20.1	601.0 (−3.8%)	59.3
TiCN + Ni	235.5 (+5.6%)	22.9	665.6 (+4.8%)	64.7

## Data Availability

The data presented in this study are available on request from the corresponding author.
